# Pantoprazole (PPZ) Inhibits RANKL-Induced Osteoclast Formation and Function In Vitro and Prevents Lipopolysaccharide- (LPS-) Induced Inflammatory Calvarial Bone Loss In Vivo

**DOI:** 10.1155/2020/8829212

**Published:** 2020-11-28

**Authors:** Yu-Xi Li, Fu-Chao Chen, Ting Liu, Zhao-Peng Cai, Keng Chen, Guo-Xue Tang, Jun-Shen Huang, Xiang-Ge Liu, Jia-Jun Huang, Peng Wang, Yu-Wei Liang, Lin Huang

**Affiliations:** ^1^Department of Orthopedics, Sun Yat-sen Memorial Hospital, Sun Yat-sen University, Guangzhou 510120, China; ^2^Department of Orthopedics, Second Affiliated Hospital of Soochow University, Soochow University, Suzhou 215000, China; ^3^Department of Anesthesia, Sun Yat-sen Memorial Hospital, Sun Yat-sen University, Guangzhou 510120, China; ^4^Department of Orthopedics, Sun Yat-sen University Eighth Affiliated Hospital, Sun Yat-sen University, Shenzhen 518033, China; ^5^Department of Ultrasound, Sun Yat-sen Memorial Hospital, Sun Yat-sen University, Guangzhou 510120, China

## Abstract

Bone remodeling is a process delicately balanced between osteoclastic bone resorption and osteoblastic bone formation. Osteoclasts (OCs) are multinucleated giant cells formed through the fusion of monocytic precursors of the hematopoietic stem cells lineage. OCs are the exclusive cells responsible for the resorption and degradation of the mineralized bone matrix. Pantoprazole (PPZ), a proton pump inhibitor (PPI), is commonly prescribed to reduce excess gastric acid production for conditions such as gastroesophageal reflux disease and peptic ulcer disease. Studies have found contradictory effects of PPI therapy on bone metabolism due to the lack of understanding of the exact underlying mechanism. In this study, we found that PPZ inhibits receptor activator of nuclear factor-*κ*B (NF-*κ*B) ligand- (RANKL-) induced osteoclastogenesis from bone marrow monocytic/macrophage (BMMs) precursors and the bone-resorbing activity of mature OCs. Correspondingly, the expression of OC marker genes was also attenuated. At the molecular level, PPZ treatment was associated with reduced activation of the ERK MAPK signaling pathways crucial to OC differentiation. Additionally, the in vivo administration of PPZ protected mice against lipopolysaccharide- (LPS-) induced inflammatory calvarial bone erosion, as a result of the reduced number and activity of OCs on the calvarial bone surface. Although PPI use is associated with increased risk of osteoporosis and bone fractures, our study provides evidence for the direct inhibitory effect of PPZ on OC formation and bone resorption in vitro and in vivo, suggesting a potential therapeutic use of PPZ in the treatment of osteolytic disease with localized bone destruction.

## 1. Introduction

Proton pump inhibitors (PPIs) such as pantoprazole (PPZ) are well-established standard treatment strategy for the treatment of upper gastrointestinal disorders such as gastroesophageal reflux disease and peptic ulcers. PPZ was developed in Germany in October 1994 and was marketed in South Africa in the same year. Pantoprazole is a third-generation proton pump inhibitor, and its efficacy and safety have been fully confirmed. PPZ is usually administered clinically by injection or oral. When administered intravenously, the usual dose of pantoprazole sodium for injection is 40 mg twice daily. The oral dosage for the treatment of peptic ulcers and related diseases is usually 40 mg once a day. PPIs irreversibly bind to the H^+^/K^+^-ATPase or the proton pumps in the apical surface of parietal cells in the stomach to inhibit acid secretion. Although regarded as safe and effective treatment options against these disorders, population-based epidemiological studies have raised concerns that long-term and high-dose use of PPIs is associated with increased fracture risks and osteoporosis [1]. However, a direct causal link has yet to be established [2–6]. In vitro animal and human data suggests that PPIs decrease the osteoclast (OC) bone resorption activity and alter the osteoblast (OB) bone-forming activity [7–12]. On the contrary, in vivo studies regarding the effects of PPIs on bone metabolism have been less conclusive [13–17]. Because of these inconclusive effects of PPIs on bone metabolism, the aim of this study was to determine the cellular effects of an important representative of PPIs, PPZ, on OC formation and function.

Bone is a dynamic tissue that undergoes constant life-long remodeling via the balanced activities of the bone-forming OBs and bone-resorbing OCs [18, 19]. Imbalances in this remodeling process predominantly favour excessive OC bone resorption. OCs participate a key role in various osteolytic conditions such as osteoporosis, tumour-associated bone destruction, periprosthetic loosening, and pathological inflammation-induced osteolysis [1, 20–25]. OC formation and subsequent bone resorption are primarily regulated by two key cytokines, macrophage colony-stimulating factor (M-CSF), and receptor activator of nuclear factor-*κ*B (NF-*κ*B) ligand (RANKL) [26]. Binding of RANKL to receptor RANK on the surface of monocytic/macrophage precursors results in the activation of a cascade of signaling pathways that culminates in precursor cell fusion into multinucleated giant cells [27]. The vacuolar H^+^-ATPases (V-ATPases) secrete acids into the space between the plasma membrane and opposing bone surface creating an acidic extracellular microenvironment, which is needed for the dissolution of underlying mineralized bone matrix [28]. It is this similarity in the acid secreting mechanism with gastric H^+^/K^+^-ATPases that the OC V-ATPases were proposed as possible targets for the action of PPIs. In addition to its role in OC bone resorption, V-ATPases have also been reported essential for OC formation [29–32].

Based on this knowledge, we thus examined the direct cellular effects of PPZ, a classical and important PPI, on OC formation and bone resorption in vitro and its potential therapeutic effects in a murine model of LPS-induced inflammatory bone loss in vivo. The findings we believe will provide further insight into the cellular effects of PPIs on OC and evidence for the therapeutic application of PPIs for the prevention and treatment of osteolytic conditions mediated by excessive OC bone resorption.

## 2. Materials and Methods

### 2.1. Media and Reagents

**Figure 1:**
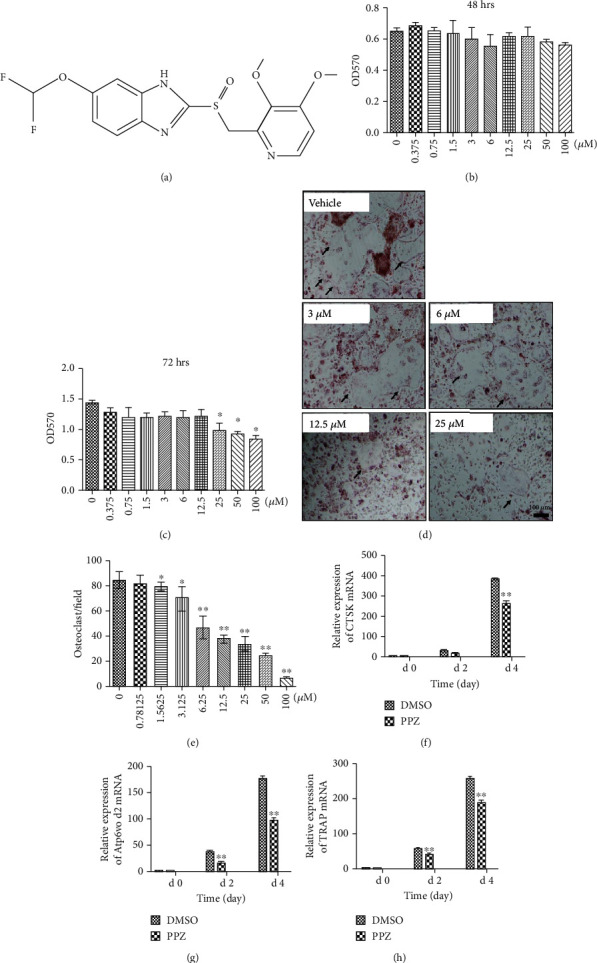
PPZ inhibits RANKL-induced osteoclast (OC) formation and suppresses RANKL-induced OC-related gene expression in vitro. (a) Chemical structure of PPZ. (b, c) Effects of PPZ on viability and proliferation of bone marrow-derived macrophages (BMMs) at 48 and 72 hrs, respectively. The absorbance of the optical density was measured at 570 nm (OD570). (d) Quantitative analysis of the numbers of TRAP-positive multinucleated (nuclei ≥ 3) cells formed in the presence of different concentrations of PPZ. (e) BMMs were cultured under RANKL stimulation with 0, 3, 6, 12.5, and 25 *μ*M of PPZ for 7 days and treated with TRAP staining (magnification: 100x; scale bar = 100 *μ*m). TRAP-positive cells with ≥3 nuclei were considered OCs (magnification: 100x; scale bar = 100 *μ*m). (f–h) Quantitative real-time PCR analyses of relative levels of OC marker genes (*TRAP*, *CTSK*, and *V-ATPase d2*) at day 0, day 2, and day 4 in the presence of PPZ (12.5 *μ*M). The expression levels of these genes were normalized to the expression of *β*-actin. Data are presented as mean ± SD; ^∗^*P* < 0.05, ^∗∗^*P* < 0.01 relative to RANKL-induced controls.

PPZ (Figure 1(a); Beyotime Biotechnology, Jiangsu, China) was dissolved in dimethylsulfoxide (DMSO) to make stock solutions. When required, PPZ stock solutions were further diluted in cell culture medium to desired concentrations so that the DMSO comprised <0.1% of the total volume. Alpha Modification-Minimal Essential Medium (*α*MEM), Dulbecco's Modified Eagle's Medium (DMEM), fetal bovine serum (FBS), and penicillin/streptomycin were purchased from Trace Scientific Ltd. (Sydney, Australia). RANKL and M-CSF were purchased from R&D Systems (Minneapolis, MN, USA). Specific antibodies against total and phosphorylated forms of ERK and *β*-actin were from Cell Signaling Technology (Danvers, MA, USA). Specific antibodies against total and phosphorylated I*κ*B*α* were purchased from Abcam (Cambridge, UK). Tartrate-resistant acid phosphatase (TRAP) enzymatic activity was detected using the Leukocyte Acid Phosphatase Staining Kit from Sigma-Aldrich (St. Louis, MO, USA). All other reagents were purchased from Sigma-Aldrich unless otherwise stated.

### 2.2. Cell Culture and OC Formation Assay

Primary bone marrow monocytes/macrophages (BMMs) were isolated from the whole bone marrow of 6-week-old male ICR mice (Institute of Cancer Research). Extracted BMMs were maintained in *α*MEM supplemented with 10% FBS, 1% penicillin/streptomycin, and 20 ng/ml M-CSF (complete *α*MEM) in a humidified incubator with 5% CO_2_ and temperature of 37°C [33]. After 2 days of culture or when cell monolayers reached 90% confluence, nonadherent cells were removed by gentle washes with phosphate-buffered saline (PBS), and the remaining adherent cells were considered BMMs and used for downstream experiments. For the OC formation assay, BMMs were seeded in 96-well plates at a density of 7 × 10^3^ cells/well in triplicates in complete *α*MEM and stimulated with 50 ng/ml RANKL without or with 3, 6, 12.5, or 25 *μ*M of PPZ. Cells treated with equal dilution of DMSO (<0.1% of the total volume) were used as untreated controls. Cell culture media without or with PPZ were replaced every 2 days for 7 days until multinucleated OCs were observed in RANKL-only-treated controls. Cells were washed with PBS, fixed in 4% paraformaldehyde (PFA) for 20 mins, and then stained with TRAP reagent and placed in the incubator for 30 mins for the TRAP activity. Observing at the magnification of 100x with an optical microscope and the number of TRAP-positive mature OCs with 3 or more nuclei in a region of interest in each of three replicate samples were quantified using ImageJ software (NIH, USA).

### 2.3. Cytotoxicity Assay

The 3-(4,5-dimethylthiazol-2-y1)-2,5-di-phenyltetrazolium bromide (MTT) assay was performed to examine the viability/proliferation of BMMs following PPZ treatment. BMMs were seeded in 96-well plates at a density of 8 × 10^3^ cells/well in triplicates in complete *α*MEM for 24 hrs. Cells were then treated with serial dilutions of PPZ (0.375, 0.75, 1.5, 3,6, 12.5, 25, 50, or 100 *μ*M) for 48 and 72 hrs. At the end of the treatment period, 10 *μ*l of MTT solution (5 mg/ml in PBS) was added to each well and incubated for a further 4 hrs at 37°C. Culture medium was then replaced with 150 *μ*l of DMSO to solubilize the purple formazan crystals. The absorbance was measured at 570 nm using an ELX800 absorbance microplate reader (BioTek Instruments, Winooski, VT, USA).

### 2.4. Podosomal-Actin Cytoskeleton Immunofluorescence

To examine the cytoskeletal podosomal-actin belt, BMM-derived OCs cultured and treated with PPZ as described above were fixed with 4% PFA for 20 mins and then permeabilized with 0.1% Triton X-100 in PBS for 5 mins at room temperature. Cells were then incubated with Acti-stain™ 488 Fluorescent Phalloidin (Cytoskeleton Inc., Denver, CO, USA) in the dark at room temperature for 30 mins. Cells were washed three times with PBS, and the nuclei were counterstained with 1 *μ*g/ml DAPI for 30 mins. Immunofluorescence images were acquired and processed using an immunofluorescence microscope.

### 2.5. Bone Absorption Assay

BMMs were seeded onto bovine cortical bone discs placed in 96-well culture plates at a density of 1 × 10^4^ cells/well in complete *α*MEM in triplicates and stimulated with 50 ng/ml RANKL in the absence or presence of serial dilutions of PPZ (1.5, 3.125, 6.25, 12.5, 25, or 50 *μ*M). Culture media were replaced every 2 days for 15 days after which cells were removed from bone discs by immersion in 6% sodium hypochlorite solution. Bone resorption pits were assessed and images acquired using the FEI Quanta 250 scanning electron microscope (Thermo Fisher Scientific, Waltham, MA, USA). The bone resorption area in each treatment sample was quantified using ImageJ software.

### 2.6. Quantitative Analysis of the Gene Expression by Real-Time PCR

Quantitative real-time PCR was employed to examine the gene expression of OC marker genes during RANKL-induced OC formation. To this end, total RNA was extracted from BMM-derived OCs treated with PPZ using TRIzol RNA extraction reagent (Thermo Fisher Scientific) according to the manufacturer's instructions. Complementary DNA (cDNA) was then reverse transcribed using 1 *μ*g of extracted RNA, 2 *μ*l of 5×PrimeScript RT Master Mix (Takara Bio, Shiga Prefecture, Japan), and 4 *μ*l of RNAse-free dH_2_O, in a total volume of 10 *μ*l. Real-time PCR was performed on the ABI Prism 7500 system (Applied Biosystems, Foster City, CA, USA) using the SYBR Green qPCR Premix (Takara Bio) in accordance with the manufacturer's protocol. The PCR reaction was performed at 95°C for 10 mins (activation); followed by 40 cycles of 95°C for 10 secs, 60°C for 20 secs, and 72°C for 20 secs (amplification); and a final extension at 72°C for 90 secs. The following specific primers based on the mouse sequences were used: *β-*actin (forward: 5′-AGC CAT GTA CGT AGC CAT CC-3′; reverse: 5′-CTC TCA GCA GTG GTG GTG AA-3′), *TRAP* (forward: 5′-TCC TGG CTC AAA AAG CAG TT-3′; reverse: 5′-ACA TAG CCC ACA CCG TTC TC-3′), *cathepsin K* (*CTSK*) (forward: 5′-CTT CCA ATA CGT GCA GCA GA-3′; reverse: 5′-TCT TCA GGG CTT TCT CGT TC-3′), and *V-ATPase V0d2* (ATP6V0d2) (forward: 5′-AAG CCT TTG TTT GAC GCT GT-3′; reverse: 5′-TTC GAT GCC TCT GTG AGA TG-3′). Quantitative real-time PCR was used to detect the expression of OC marker genes (TRAP, CTSK, and V-ATPase d2) at day 0, day 2, and day 4 of RANKL stimulation without or with PPZ treatment. *β*-Actin was used as internal loading control and normalized. The expression levels of these genes were normalized to the expression of *β*-actin and were analyzed using the 2^−ΔΔCt^ method. Data are presented as mean ± SD: ^∗^*P* < 0.05, ^∗∗^*P* < 0.01, ^∗∗∗^*P* < 0.001 relative to RANKL-induced controls. Forward and reverse primers for each gene are available upon request from the corresponding authors.

### 2.7. Western Blot Analysis

To examine the long-term signaling response to PPZ, BMMs were cultured and supplemented with M-CSF (30 ng/ml) and RANKL (50 ng/ml). BMMs were treated in the presence or absence of PPZ for 0, 1, 3, and 5 days, and the total protein of these time points was obtained, respectively. To examine early RANKL-induced signaling responses, total cellular proteins (TCPs) were extracted using RIPA lysis buffer (Sigma-Aldrich) from BMMs pretreated with 12.5 *μ*M of PPZ for 1 hr followed by stimulation with 50 ng/ml of RANKL for 10, 20, 30, or 60 mins. BMM cells treated with RANKL only for the same time period were used as positive controls. Afterward, cell lysates were centrifuged at 12,000 × *g* for 15 mins at 4°C, the supernatants containing TCPs were collected, and protein concentrations were quantified using the BCA Protein Assay Kit (Thermo Fisher). Thirty micrograms of extracted proteins was resolved on 10% SDS-PAGE gel, and separated proteins were then transferred to PVDF membranes (Bio-Rad Laboratories, Hercules, CA, USA) overnight at 4°C. Membranes were blocked with 5% (*w*/*v*) nonfat dry milk in TBST (Tris-buffered saline with 0.1% Tween-20) at room temperature for 1 hr and then incubated with specific primary antibodies (diluted 1 : 1000 in 1% (*w*/*v*) nonfat dry milk in TBST) overnight at 4°C. After incubation with primary antibodies, membranes were washed three times with TBST and then incubated with appropriate HRP-conjugated secondary antibodies (diluted 1 : 1000 in 1% (*w*/*v*) nonfat dry milk in TBST; Sigma-Aldrich) for a further 2 hrs at room temperature. Protein bands were visualized using a LAS-4000 Science Imaging System (Fujifilm, Tokyo, Japan) following exposure to ECL chemiluminescence substrate, and acquired images were analyzed with ImageJ software. *β*-Actin was used as internal loading control and normalized; then, this was followed by quantitative measurement of the relative expressions of pERK/ERK.

### 2.8. Murine Model of LPS-Induced Inflammatory Calvarial Osteolysis

LPS-induced inflammatory calvarial osteolysis in mice was established as previously described [34–37]. All animal procedures and experiments were approved by the Ethics Committee of the Animal Experiment Center and performed in accordance with the guidelines for animal experimentation of the Institute Committee of Sun Yat-sen University. The mice were housed in ambient temperature of 22°C to 24°C and humidity-controlled environment of 55% to 60% with a 12 hr light/dark cycle. Mice were given ad libitum access to standard rodent chow and clean drinking water. Briefly, 40 male 8-week-old ICR mice were randomly assigned to four groups (*n* = 10 mice each group): sham (injection of PBS), LPS (injection of 5 mg/kg LPS and PBS), low-dose PPZ (injection of 5 mg/kg LPS and 2.5 mg/kg PPZ), and high-dose PPZ (injection of 5 mg/kg LPS and 10 mg/kg PPZ). The day before the commencement of LPS injection, mice received either subcutaneous injections of PBS or PPZ injections (prophylactic treatment) under the periosteum towards the sagittal midline suture of the calvarium under light anesthesia. The next day, LPS was subcutaneously injected to the same area near the midline suture of the calvarium. PBS or PPZ injections were carried out every other day over a 7-day period. At the end of the experimental period, all mice were sacrificed, and the calvaria were excised, fixed in 4% PFA for 2 days, and then processed for microcomputed tomography (*μ*CT) and histological assessment.

### 2.9. Microcomputed Tomography (*μ*CT) Scanning

The fixed calvarial bones were scanned and analyzed using a high-resolution micro-CT scanner (SkyScan 1178; Bruker, Aartselaar, Belgium) at an isometric voxel resolution of 18 *μ*m, X-ray voltage of 80 kV, and current of 100 *μ*A. Three-dimensional reconstructions of the calvarial bones were carried out using SkyScan NRecon software with the cone beam volumetric algorithm (Bruker). A square volume of interest (VOI; 3 × 3 × 1 mm) centered around the midline suture was selected for quantitative bone morphometric analyses of percentage of bone volume to tissue volume (BV/TV, %), the number of porosities, and percentage of total osteolytic lesions for each sample using SkyScan CTan software (Bruker).

### 2.10. Histological and Immunohistochemical Analysis

Following *μ*CT analyses, the fixed calvarial bones from each treatment group were decalcified in 10% EDTA for 14 days at 4°C and then embedded in paraffin. Histological sections of 4 *μ*m thickness were prepared for hematoxylin and eosin (H&E) and TRAP staining as previously described [38]. We used xylene, ethanol, PBS, hematoxylin, and eosin to stain and dehydrate according to certain steps. Stained sections were imaged using a high-quality light microscope. Image ProPlus 6.0 software (Media Cybernetics, Rockville, MD, USA) was used to assess histomorphometric bone parameters of the area of erosion (mm^2^), the number of TRAP-positive OCs, and the OC surface per bone surface for each sample.

### 2.11. Statistical Analysis

All experimental data are presented as mean ± standard deviation (SD) from at least three independent experiments and three parallel holes in each experiment (*n* = 9). Differences between experimental and control groups were evaluated by Student's *t*-test. Results for multiple group comparisons were analyzed by Scheffe's test and one-way analysis of variance (ANOVA) with the SPSS 19.0 software (SPSS Inc., Chicago, IL, USA). *P* value less than 0.05 (^∗^*P* < 0.05, ^∗∗^*P* < 0.01, and ^∗∗∗^*P* < 0.001) was considered statistically significant.

## 3. Results

### 3.1. PPZ Inhibited RANKL-Induced OC Formation In Vitro

The potential cytotoxic effects of PPZ (Figure 1(a)) on BMM cell viability were firstly assessed. BMMs were exposed to serial dilutions of PPZ starting from 100 *μ*M down to 0.375 *μ*M for 48 and 72 hrs after which cell viability/proliferation was evaluated by the MTT assay. The results showed that BMM viability was not affected by PPZ at concentrations of up to 12.5 *μ*M in a 72-hour experiment (Figures 1(b) and 1(c)). We next examined the effects of PPZ on RANKL-induced OC formation. To this end, BMMs were stimulated with RANKL for 7 days in the absence or presence of indicated concentrations of PPZ and then stained for the TRAP activity. As compared to RANKL-only-treated controls, PPZ dose-dependently inhibited the formation of well-spread TRAP-positive multinucleated OCs (Figures 1(d) and 1(e)). These data indicate that PPZ can directly impact OC formation in an inhibitory and dose-dependent manner.

### 3.2. PPZ Suppressed the OC Marker Gene Expression

RANKL-induced OC formation is associated with the upregulation of genes that are involved in precursor cell fusion and bone resorption, often referred to as OC marker genes [39]. Thus, to confirm the inhibitory effect of PPZ on OC formation, we examined the expression of *ATP6V0d2* encoding the V-ATPase V0 domain subunit d2 implicated in precursor fusion and *cathepsin K* (*CTSK*) and *TRAP* both of which are required for the OC bone resorptive function, by real-time PCR. As shown in Figures 1(f)–(h), the expression of *ATP6V0d2*, *CTSK*, and *TRAP* genes in control cells were markedly upregulated in response to RANKL in a time-dependent manner. On the other hand, treatment with PPZ (12.5 *μ*M) significantly hindered the expression these genes, which corroborates with the inhibitory cellular effect on OC formation observed earlier. Together, the data confirmed the antiosteoclastic effect of PPZ in vitro.

### 3.3. PPZ Impaired OC Precursor Cell Fusion and Cell Spreading

**Figure 2:**
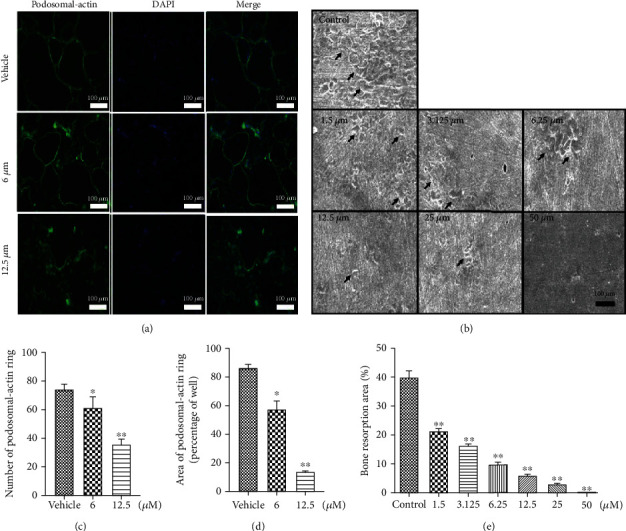
PPZ attenuates podosomal-actin ring formation and bone resorption in vitro. (a) BMMs were stimulated by RANKL at 6 and 12.5 *μ*M of PPZ and stained with podosomal-actin and DAPI, respectively (magnification: 100x; scale bar = 100 *μ*m). (b) BMMs were cultured on bone slices and stimulated by M-CSF and RANKL in the presence of different concentrations of PPZ. After 15 days, bone resorption lacunae were observed by scanning electron microscopy (magnification: 100x; scale bar = 100 *μ*m). (c, d) Quantitative measurement of the size and number of podosomal-actin ring per well using ImageJ. (e) Resorption pit area measurements. The area of bone resorption was quantitated using ImageJ. Data are presented as mean ± SD; ^∗^*P* < 0.05, ^∗∗^*P* < 0.01 relative to RANKL-induced controls.

Considering that PPZ inhibits RANKL-induced OC formation and marker gene expression, we thus further examined the effect of PPZ on actin cytoskeleton. The formation of the unique actin-rich adhesive cytoskeletal structures, the podosomal-actin belt (Figure 2(a), controls), is necessary for cell spreading during OC formation and efficient OC bone resorption, respectively (Figure 2(b), controls). As shown in Figure 2(a), the addition of PPZ (12.5 *μ*M) to cultures of BMM-derived OCs cultured on plastic affected the formation of the podosomal-actin belt circumscribing the periphery of the cell, and the size of the podosomal-actin belt was significantly smaller than control OCs indicating reduced cell spreading (Figure 2(a)). This finding suggests that PPZ inhibits OC formation by impairing OC spreading. This is in line with the decrease gene expression of ATP6V0d2 observed above.

### 3.4. PPZ Attenuated OC Bone Resorption In Vitro

Bone resorption is the primary function of OCs (Figure 2(b), controls); thus, we next examined the effects of PPZ on the bone resorption activity of OCs in vitro. BMM-derived OCs cultured on bovine bone discs were treated with indicated concentrations of PPZ after which bone resorption pits were assessed by SEM. Compared with RANKL-only-treated OCs which effectively resorbed the mineralized bone matrix and leaving behind resorption pits and trails, OCs treated with PPZ demonstrated a dose-dependent decrease in the bone resorption activity (Figures 2(b) and 2(e), treatment panels). Quantitative analysis of the resorbed area relative to the total bone surface showed dramatic reduction in resorption as compared to RANKL-treated only controls (Figure 2(e)). Based on these findings, we can conclude that PPZ also exerts antiresorptive effects on OC bone resorption in vitro.

### 3.5. PPZ Treatment Was Associated with Decreased Activation of ERK MAPK Signaling Pathways in Response to RANKL

**Figure 3:**
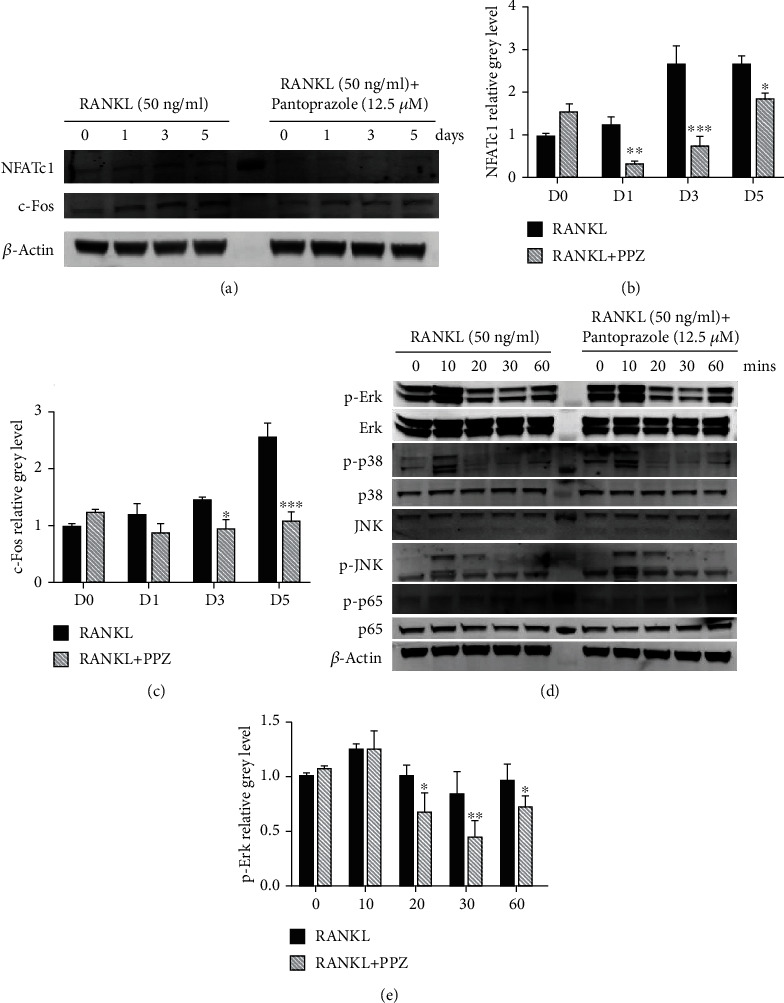
PPZ suppresses osteoclastogenesis by inhibiting the MAPK pathway and phosphorylation of Erk. (a) BMMs were cultured with M-CSF and RANKL, with or without PPZ (12.5 *μ*M) for 0, 1, 3, and 5 days. The cell lysates were probed for protein levels using western blot analysis. (b) PPZ treatment suppressed the expression of NFATc1. (c) PPZ treatment suppressed the expression of c-Fos. (d) BMMs were pretreated with 12.5 *μ*M PPZ for 1 h and then stimulated with RANKL for indicated times. The cell lysates were probed for protein levels using western blot analysis. (e) The expressions of pERK relative to total ERK was determined using ImageJ software. Data are presented as mean ± SD; ^∗^*P* < 0.05, ^∗∗^*P* < 0.01 relative to RANKL-induced controls.

To investigate whether PPZ has the inhibitory effect on RANKL-induced osteoclastogenesis, we investigated the expression of NFATc1 and c-Fos, which were the key transcription factors for osteoclast differentiation. The results of western blotting showed that the expression of NFATc1 and c-Fos increased gradually over time, staying in its peak after 3-day treatment of RANKL (Figures 3(a)–(c)). However, the expression level of NFATc1 and c-Fos in the PPZ-treated group reduced significantly relative to the control group, indicating the inhibitory effect of PPZ on osteoclastogenesis.

Furthermore, RANKL-induced OC formation, gene expression, and bone resorption are dependent on the activation of key signaling pathways in which the early activation of NF-*κ*B and MAPK in response to RANKL plays essential roles. Thus, we examined the effect of PPZ on these signaling cascades in an attempt to determine the molecular mechanisms underlying PPZ inhibitory effects. To this end, western blot analyses were performed on TCPs extracted from BMMs pretreated with PPZ for 1 hour prior to stimulation with RANKL for 10, 20, 30, and 60 mins. In untreated cells, ERK was rapidly phosphorylated within 10 mins of RANKL stimulation, persisting for 10 mins before it was dephosphorylated back down to baseline levels (Figures 3(d) and 3(e)). Again, treatment with PPZ was associated with reduced ERK phosphorylation (Figures 3(d) and 3(e)). Moreover, it was observed that the phosphorylation of p38 and JNK was activated in the control groups, while no significant inhibitory effect was observed in the PPZ-treated groups. Taken together, it appears that treatment of BMM with PPZ is associated with reduced activation of MAPK signaling cascades in response to RANKL, and this in part can result in attenuation of OC formation.

### 3.6. PPZ Prevents LPS-Induced Inflammatory Calvarial Bone Loss In Vivo

**Figure 4:**
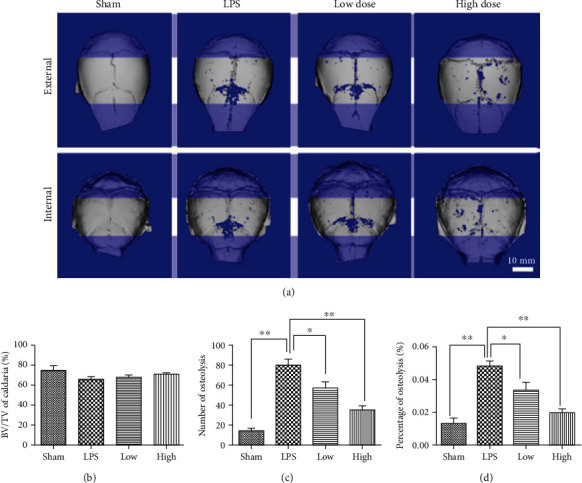
PPZ protects against lipopolysaccharide- (LPS-) induced bone loss in vivo. (a) Micro-CT scanning and 3D reconstruction of the entire calvaria of mice from the sham group (PBS), LPS group (LPS 5 mg/kg body weight), LPS with low-dose PPZ group (2.5 mg/kg), and LPS with high-dose PPZ group (10 mg/kg) (*n* = 10). (b) Quantitative analysis of bone volume/total volume (BV/TV) of calvaria (*n* = 10). (c, d) Number and percentage of osteolytic lesions (*n* = 10). Data are presented as mean ± SD; ^∗^*P* < 0.05, ^∗∗^*P* < 0.01 relative to control.

**Figure 5:**
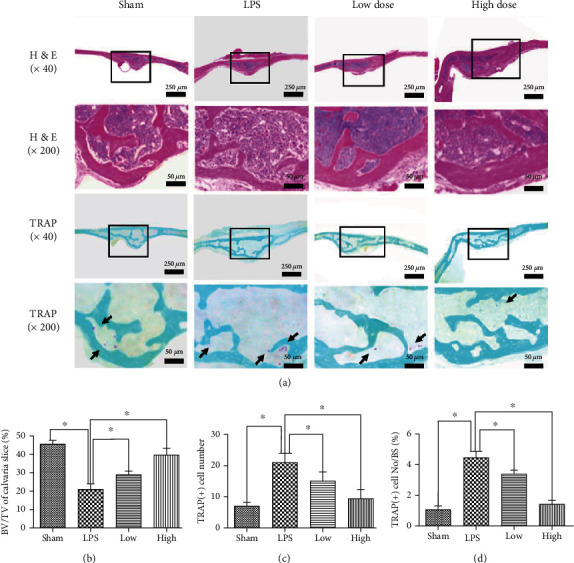
Histological and histomorphometric analysis of the effect of PPZ on LPS-induced bone loss in vivo. (a) Representative images of calvaria stained with H&E and TRAP from the sham group (PBS), LPS group (5 mg/kg bodyweight), LPS with low-dose PPZ group (2.5 mg/kg bodyweight), and LPS with high-dose PPZ group (10 mg/kg bodyweight). Black arrows indicate TRAP-positive cells. (b–d) Quantification of BV/TV of calvaria slice (%), TRAP-positive cell number, and TRAP-positive cell no./BS (*n* = 10). Data are presented as mean ± SD; ^∗^*P* < 0.05, ^∗∗^*P* < 0.01 relative to control.

Finally, we examined whether the in vitro inhibitory effect of PPZ on OC formation and bone resorption can be observed in an in vivo model of OC-mediated bone loss. To this end, we employed the murine model of LPS-induced inflammatory calvarial osteolysis, a condition of elevated OC bone destruction due to the LPS-induced inflammatory response. Eight-week-old ICR mice were given subcutaneous injections of PBS or PPZ over the sagittal midline suture of the calvarium 1 day before LPS injection (prophylactic treatment). Seven days after the LPS injection, calvarias from each treatment group were excised and analyzed by *μ*CT scanning. Three-dimensional reconstructions revealed pronounced bone erosion in LPS-treated groups compared to sham controls (Figure 4(a)). Quantitative morphometric analyses further showed significant reductions in bone volume (Figure 4(b)) and marked increases in calvarial osteolytic lesions (Figures 4(c) and 4(d)). Prophylactic treatment with PPZ dose-dependently mitigated the severity of LPS-induced bone erosion (Figure 4(a)) and reduced osteolytic lesions (Figures 4(c) and 4(d)). The protective effect of PPZ on LPS-induced bone erosion was further confirmed by histological assessments. In contrast to the LPS-treated group which shows a strong OC-mediated bone erosion, PPZ treatment significantly reduced the number of TRAP-positive OCs found in the bone (Figure 5(a)). As with morphometric analysis, histomorphometric analysis of bone volume showed restoration of bone volume in the presence of PPZ (Figure 5(b)) as a result of decreased TRAP-positive OC number and the number of OC per bone surface (OC/BS) (Figures 5(c) and 5(d)). Taken together, we have shown that PPZ administration can protect mice against LPS-induced osteolytic bone loss in vivo.

## 4. Discussion

Elevated OC formation and/or bone resorptive activity leads to excessive bone destruction, a characteristic feature of many osteolytic bone diseases. OC bone resorption relies on the V-ATPase proton pump to acidify the extracellular resorption space for the dissolution and degradation of the underlying mineralized bone matrix. In this study, we investigated the effect of the classical and important proton pump inhibitor (PPI), namely, PPZ, on OC formation and bone resorption in vitro and the potential therapeutic effect of PPZ administration against LPS-induced inflammatory osteolysis in mice in vivo. Despite conflicting data for the effects of PPI on bone metabolism in the literature, the results in our study suggest an antiosteoclastic/antiresorptive effect for PPZ with potential therapeutic application against conditions of excessive OC-mediated bone destruction.

Using in vitro cell-based assays, we demonstrated that PPZ exerts direct inhibitory actions against RANKL-induced OC formation, marker gene expression, and bone resorption. OC differentiation and formation are a multistep process that involves cell proliferation, precursor fusion, and subsequent activation towards bone resorption [39]. This process is highly dependent on the activation of key signaling pathways in response to RANKL binding to receptor RANK on OC precursors. The earliest signaling pathways activated include the NF-*κ*B and MAPK (ERK, p38, and JNK) signaling cascades [40]. RANKL binding induces the rapid phosphorylation and degradation of I*κ*B*α* consequently enabling NF-*κ*B p65 phosphorylation and nuclear translocation. At the same time, the three MAPK family members ERK, p38, and JNK are also activated by phosphorylation, and together with NF-*κ*B, they transcriptionally activate target genes that are involved in OC formation, activation, and survival [27]. In our study, we found that PPZ was associated with a reduced phosphorylation of ERK, thereby impairing the activation of the ERK MAPK signaling cascades, respectively. Consistent with attenuated MAPK signaling, the transcriptional induction of OC marker genes such as *TRAP*, *CTSK*, and *ATP6V0d2* was also downregulated by PPZ. Given that PPZ inhibited ERK MAPK pathways, it is suggested that PPZ could target a common upstream effector for these pathways which requires further investigation to identify.

In addition to antiosteoclastogenic effects, PPZ exerted antiresorptive effects against OC bone resorption. PPZ dose-dependently inhibited the bone resorptive activity of OCs; however, the underlying mechanism requires more in-depth investigations. As suggested earlier, PPIs might inhibit the V-ATPase proton pump found in a ruffled border of resorbing OCs. However, two studies which investigated the impact of short-term (14-days) and long-term (12-weeks) PPI administration for the osteoclastic proton pump showed no measurable effects on biochemical markers of calcium and bone metabolism [41, 42]. On the contrary, an in vivo study by Joo et al. found that a low-calcium diet coupled with PPI administration altered the OC activity and bone resorption signaling [13]. This is in line with our current data as well as studies by Tuukkanen et al. [9], Mizunashi et al. [10], Sheraly et al. [43], and Prause et al. [11]. Further, studies are required to better define the mechanism by which PPIs inhibit or alter OC bone resorptive functions, and this may include looking into specific functional acidification assays to see if PPIs such as PPZ can directly inhibit the OC V-ATPase proton pump.

Having established promising in vitro effects of PPZ on OC formation and activity, we then explored the potential therapeutic benefits of PPZ administration against OC-mediated bone destruction in vivo. To this end, a murine model of LPS-induced inflammatory calvarial osteolysis was used. LPS is an endotoxin and a component of the outer membrane of Gram-negative bacteria that rapidly induces bone loss through the stimulation of OC formation and bone resorption via the activation of the NF-*κ*B and MAPK signaling cascades in macrophages and monocytes [44, 45]. Prophylatic treatment with PPZ reduced the severity of LPS-induced OC-mediated bone destruction in a dose-dependent manner. PPZ administration greatly reduced the TRAP-positive OC number in the bone as well as the number of OC per bone surface, indicative of reduced active bone resorption. This protective effect of PPZ against LPS-induced bone loss could be attributed to the antiosteoclastogenic and antiresorptive effects of PPZ that we observed in vitro. However, our current data does not allow us to exclude the possibility that PPZ might affect OB bone formation as PPIs have been shown to alter the OB activity [46, 47]. Thus, further studies will need to be conducted to examine the effects of PPZ on OB function. Moreover, studies showed that long-term usage of PPIs may induce acid inhibition-related adverse events like pneumonia and gastrointestinal infection [48].

## 5. Conclusion

The findings in this study highlighted the direct inhibitory effect of PPZ on OC formation, gene expression, and bone resorption in vitro and protective effects against pathological OC-mediated bone loss induced by LPS in vivo. The current work offered further insights into the direct effects of PPIs on OC formation and activity and also provides evidence for the potential therapeutic applications of PPZ against localized inflammatory disease involving OC-mediated bone resorption, and possibly other localized osteolytic conditions often observed in the clinical settings.

## Data Availability

The data used to support the findings of this study are included within the article.
